# Protection of soil carbon within macro-aggregates depends on intra-aggregate pore characteristics

**DOI:** 10.1038/srep16261

**Published:** 2015-11-06

**Authors:** Alexandra N. Kravchenko, Wakene C. Negassa, Andrey K. Guber, Mark L. Rivers

**Affiliations:** 1 Department of Plant, Soil and Microbial Sciences, Michigan State University, East Lansing, Michigan, United States of America; 2 IASS-Global Soil Forum, Institute for Advanced Sustainability Studies, Potsdam, Germany.; 3 Center for Advanced Radiation Sources, The University of Chicago, Argonne National Lab, Argonne, Illinois, United States of America

## Abstract

Soil contains almost twice as much carbon (C) as the atmosphere and 5–15% of soil C is stored in a form of particulate organic matter (POM). Particulate organic matter C is regarded as one of the most labile components of the soil C, such that can be easily lost under right environmental settings. Conceptually, micro-environmental conditions are understood to be responsible for protection of soil C. However, quantitative knowledge of the specific mechanisms driving micro-environmental effects is still lacking. Here we combined CO_2_ respiration measurements of intact soil samples with X-ray computed micro-tomography imaging and investigated how micro-environmental conditions, represented by soil pores, influence decomposition of POM. We found that atmosphere-connected soil pores influenced soil C’s, and especially POM’s, decomposition. In presence of such pores losses in POM were 3–15 times higher than in their absence. Moreover, we demonstrated the presence of a feed-forward relationship between soil C decomposition and pore connections that enhance it. Since soil hydrology and soil pores are likely to be affected by future climate changes, our findings indicate that not-accounting for the influence of soil pores can add another sizable source of uncertainty to estimates of future soil C losses.

Soil is a key component of terrestrial ecosystems that contains almost twice as much carbon (C) as atmosphere; and it is the C that can be easily lost under right environmental settings^1^,^2^. Thus, understanding the mechanisms of C protection in soil is imperative for mitigation of climate change effects.

Lately, the concept of physical protection as the key driver of soil C’s fate has gained wide recognition^3^,^4^,^5^. Physical protection is enabled by inaccessibility of soil C to microbial decomposers and their enzymes^4^. At micro-scales soil consists of diverse micro-environments generating a wide range of conditions for C accessibility to microbial decomposers and for microbial functioning^6^. At a scale of a few microns (<10 μm) soil textural and mineralogical characteristics, e.g., presence of clay and its minerology, play an important role in driving physical protection by affecting organic matter interactions with soil minerals^7^. However, at a 10–1000 μm scale, soil C protection becomes largely a function of a physical disconnect between the organic matter and its decomposers. Thus, at this scale C protection is governed by soil structure, a.k.a. abundance, spatial arrangements, and characteristics of soil pores^8^,^9^,^10^. Soil aggregates, the building blocks of soil structure, and their internal pore characteristics, is what creates the physical micro-environments enabling or disabling connections, and thus plays a major role as both an arena and a product of soil C stabilization and dynamics^11^,^12^.

In order to understand, quantify, and model processes involved in physical protection it is necessary to look at them at the scale at which they occur^13^, that is, at a micro-scale. Even though conceptual understanding has been attained, the exact mechanisms enabling physical protection remain insufficiently lucid for their quantitative representation in process-based models; the crucial tools for assessing C cycle under future climate and for developing climate change mitigation strategies^7^. It is expected that changing climate will intensify water cycle in temperate regions^14^. Resultant greater frequencies of soil drying-wetting events will affect soil pore formation and characteristics. How changes in soil pores will influence protection of soil C is unclear, contributing yet another unknown to assessments of future C cycle.

At present, majority of soil physical protection research focuses on a concept of protection via intra-aggregate occlusion^15^. While of great value to facilitate general understanding, this concept does not allow explicit accounting for the actual mechanics of the accessibility process, which is driven by pore network characteristics. In order to be able to quantify and model processes involved in physical protection it is crucial to study the soil with the actual pore structure, i.e., its micro-environmental conditions, being intact^16^.

The component of major importance for soil C processes is particulate organic matter (POM). Particulate organic matter consists of undecomposed remnants of plants and animals and is regarded as most labile component of the soil organic matter^17^. It is also the component that is most sensitive to changes in soil use and management and, moreover, the component that can be easily lost or likewise easily protected under right micro-environmental conditions^17^. Carbon from POM can constitute a substantial portion, i.e., 5–15%, of soil organic C^18^, which on the global scale translates into as much as 75–230 Pg of highly decomposition conditions sensitive POM C stored in soil.

Soil C processes are known to be greatly affected by levels of soil disturbance, diversity of plant inputs, and duration of live vegetation cover^19^. Thus we explored the role of soil pores in protecting C in soils under contrasting disturbance and vegetation settings: (i) conventionally tilled and managed agricultural soil in a row crop rotation (CT); (ii) organically managed agricultural soil with winter cover crops added to the rotation (CT-cover); and (iii) soil from the land converted to native succession vegetation (NS). The practices range from high soil disturbance with minimal plant diversity and minimal duration of vegetative soil cover (CT) to high soil disturbance but greater plant diversity, and nearly continuous vegetative soil cover (CT-cover) to no soil disturbance with maximum plan diversity and continuous vegetative soil cover (NS).

To determine the role of pore characteristics in decomposition of soil organic matter, we conducted long term (120 day) incubations of intact soil macro-aggregates (n = 30), 4–6 mm in size. The aggregates were subjected to X-ray computed micro-tomography (μCT) scanning before and after the incubation (Fig. 1a). The aggregates used in the study were representative bodies of the intact soil matrix. As such, they offered an averaging compromise between enhanced C protection reported to take place within soil micro-aggregates^15^ and a lack of C protection in un-aggregated soil. The size of the studied samples enabled us to combine high resolution (13 μm) μCT scanning with high quality soil respiration measurements. The incubations were conducted at soil moisture and temperature settings optimal for organic matter decomposition^20^. Detailed examinations of the pore characteristics and changes in them as a result of the incubation were performed in each individual aggregate, a.k.a. intact soil sample (Fig. 1b).

We focused on (i) the cumulative amount of CO_2_-C emitted during the incubation as an indicator of the gross heterotrophic activity of soil microorganisms and on (ii) decomposition of POM. Using X-ray images we determined locations and sizes of POM fragments within the intact soil samples^21^, the pore characteristics associated with POM, and the changes in POM and pore characteristics that took place during the incubation (Fig. 1c, Supplementary information Movie 1).

We hypothesized that greater presence and greater connectedness of soil pores will lead to greater decomposition of intra-aggregate organic matter, including overall soil organic matter, as reflected by CO_2_-C emissions, as well as POM. Previously published works^22^,^23^,^24^,^25^ indicated that presence of medium sized pores (15–60 μm) can be particularly detrimental to soil organic matter protection, as such pores are the places of greater microbial abundance and activity^23^,^24^ and of greater organic matter decomposition^25^. Thus we also hypothesized that especially substantial losses in the studied intact samples will be associated with a greater presence of such pores.

## Results

### Particulate organic matter and its changes during incubation

The overall contents of POM and of POM of root origin in the samples from the studied practices followed the order of NS ≥ CT-cover > CT (p < 0.05) (Table 1). The same pattern for the three studied practices was also observed for the total soil C and for the cumulative amount of CO_2_-C emitted during the 120 day incubations (Table 1).

Decomposition of POM contributed to the overall decomposition process, as POM losses were positively associated with the cumulative amount of CO_2_-C emitted during the incubation (Fig. 2). Numeric positive trends were present in all three practices, while the associations between POM C losses and emitted CO_2_-C were statistically significant in CT and NS (p < 0.1). The estimated amount of POM C lost from the intact soil samples ranged from 7 to 30% of the cumulative emitted CO_2_-C (Table 1). The POM C losses were significantly higher in CT-cover than in CT and NS practices (p < 0.05).

Whether or not POM fragments would decompose within an intact soil sample, and thus contribute to the CO_2_ emission, was related to presence/absence of connections between POM and the outside atmosphere (Fig. 3). In all three practices the losses of POM fragments connected to the atmosphere were significantly greater than zero (p < 0.05). The losses of POM not connected to the atmosphere were either negligible, as in CT and NS practices, or low as in the CT-cover practice.

Across all studied aggregates POM losses during the incubation were positively correlated with the percent of atmosphere-connected >13 μm pores (*R*^2^ = 0.14, p < 0.05) (Fig. 4a). No differences could be distinguished between the relationships of POM losses vs. atmosphere-connected pores in individual practices, thus the data from all three practices were combined in a single regression analysis.

### Pores and their changes during incubation

The three studied practices differed in terms of total porosity, presence of pores  < 13 μm, and in presence and size distribution of pores >13 μm (Table 2). The aggregates from the two conservational practices, i.e., NS and CT-cover, had higher total porosity than the conventionally managed CT practice (p < 0.05). The undisturbed native vegetation NS practice had significantly higher percent of small pores ( < 13 μm) than the two agricultural practices, i.e., CT and CT-cover (p < 0.05). NS had significantly greater abundance of 13–32 μm pores than CT practice (p < 0.05). CT-cover had significantly greater abundance of >84 μm pores than CT and NS practices.

Incubation led to an overall increase in >13 μm pores in CT-cover and NS practices, while the percent of such pores did not change significantly in the CT practice (p < 0.05). Particularly substantial increases were observed in pores of 13–58 μm range (Table 2). In all three studied practices we observed an increase in presence of 13–32 μm pores (p < 0.05). Presence of 32–58 μm pores increased in CT-cover and NS, and presence of 58–84 μm pores increased in the CT-cover practice (p < 0.05).

Noticeable increases in the atmosphere-connected pores were observed only in two of the studied CT aggregates, while in the majority of CT-cover and NS aggregates presence of such pores increased significantly (Fig. 4b). Across all three practices the change in the presence of atmosphere-connected pores was positively associated with the POM losses during the incubation (*R*^2^ = 0.21, p < 0.05) (Fig. 4b). That is, the greater amount of POM was lost during incubation the greater was an increase in presence of atmosphere-connected pores.

### Associations between pores and CO2-C emissions during the incubation

The pore network characteristics that influenced CO_2_-C emission differed in NS, the practice where soil was not disturbed for >25 years, compared to soils of the annually plowed CT and CT-cover practices. Specifically, in NS the presence of atmosphere-connected medium-sized pores (32–58 μm) was positively correlated with emitted CO_2_-C (*R*^2^ = 0.35, p < 0.05) (Fig. 5a). However, no such associations were observed in samples from CT and CT-cover practices.

Moreover, in NS samples, the magnitude of decomposition processes itself affected creation of new atmosphere-connected 32–58 μm pores (Fig. 5b). The greater were the amounts of CO_2_-C emitted during the incubation the greater was the increase in occurrence of atmosphere-connected 32–58 μm pores (*R*^2^ = 0.41, p < 0.05) (Fig. 5b).

In both agricultural management practices, i.e., CT and CT-cover, the presence of small <13 μm pores appeared to be important in defining the overall decomposition (Fig. 6). Positive associations between presence of such pores and the cumulative CO_2_-C emissions were observed in both CT (*R*^2^ = 0.45, p < 0.05) and CT-cover (*R*^2^ = 0.41, p < 0.05). No such correlation was observed in NS samples.

## Discussion

The study findings supported our hypothesis that greater presence and greater connectedness of soil pores will lead to greater decomposition of intra-aggregate organic matter, reflected by both CO_2_-C emissions and by losses of POM. However, land use and management history affected the strength and magnitude of the pore-decomposition relationships.

Across all three studied practices, POM connected to the atmosphere by pores >13 μm in diameter lost 5–15% of its volume, while POM without such connections remained effectively intact in most of the studied samples (Fig. 3). The greater was decomposition of POM during the incubation the larger was a subsequent increase in presence of >13 μm pores, especially, of the atmosphere-connected pores. This trend was most pronounced in the practices with greater plant diversity and longer vegetation coverage, i.e., CT-cover and NS (Figs. 4 and 5, Table 2). These are also the practices that over-time retained and even accumulated soil C^26^,^27^, and tended to have greater POM contents and greater percent of POM represented by plant root residues (Table 1). On the other hand, in practices subjected to regular mechanical disturbance by tillage, i.e., CT and CT-cover, the presence of small  < 13 μm pores appeared to be important in defining the overall C decomposition (Fig. 6).

As expected, the overall CO_2_-C emissions and POM decomposition were linked to each other. It is likely that certain amount of C from the decomposed POM moved into and remained in the adjacent soil as hydrophilic intermediate products of decomposition^28^. However, positive association between POM losses and the cumulative emitted CO_2_-C indicates that at least some of the POM in the intact soil samples decomposed completely and was lost to atmosphere as CO_2_ (Fig. 2).

These observations point to presence of a positive feedback mechanism in contributions of >13 μm pores to protection vs. decomposition of soil C, specifically soil POM (Fig. 5). The more atmosphere-connected POM is, the more it will decompose, and greater decomposition produces yet greater connectedness. Large pores provide greater oxygen supply to ensure more complete decomposition of the organic material, while greater air diffusion through the large pores results in a faster escape of the generated CO_2_. Gases produced during decomposition create yet more pores on their escape^8^,^29^,^30^,^31^, in particular, medium-sized pores of 13–58 μm range. Pore forming root activities and greater POM contents in soils under prolonged live vegetation, i.e., in CT-cover and NS practices, apparently enhance the magnitude of these processes.

Another possible contributor to the pore-C decomposition feedback mechanism is presence of non-particulate labile C in soil matrix. Soils under long term native vegetation not subjected to mechanical disturbance, i.e., NS, contain substantial quantities of it^32^. Formation of medium-sized pores in a course of incubation likely increased access of microbial decomposers to such labile C sources, still further speeding up soil organic matter decomposition (Fig. 5). Importance of pores of this size range (e.g., 32–58 μm) for soil C processes has been demonstrated before^22^,^25^, both in terms of serving as conduits for fluxes^9^ and as environments preferred by certain microbial communities^23^. Our results indicate that in undisturbed soils of native vegetation systems such pores can serve as effective avenues of greater C losses.

Implementation of X-ray μCT tools and reliance on structurally intact soil samples enabled us to generate a new perspective on significance of pore characteristics for soil C protection; the perspective that at present is overlooked because of traditional experimental focus on structurally disturbed (ground and sieved) soil samples.

It should be noted that pores are only one of the many influences affecting C processes within each intact aggregate. Nature, origin, and decomposition status of POM are known to play a very important role in the rates of its decomposition and protection^33^. For example, a POM fragment of pyrogenic origin has a much greater resistivity to decomposition than a POM fragment originated from recently added plant residue^34^. Unfortunately, as of now there are no effective experimental tools for *in situ* determination of POM nature and origin in intact soil samples. Thus, given a wide range of factors potentially contributing to POM decomposition and CO_2_ emission in the intact soil samples of this study, it would be unrealistic to expect strong correlations between these variables and any contributing factor individually. Even though the relationships with pore characteristics observed in our study are not strong numerically, the fact that they are statistically significant and are consistent with our ad-hoc postulated research hypotheses provides a solid backing to the main conclusions of this study on the role of pores in soil C protection and decomposition.

Our findings show that accounting for the contribution of pore connectivity is particularly important for evaluating the fate of soil POM. Based on our results, a conservative estimate is that approximately 10% of soil POM is currently being protected due to lack of pore accessibility. This constitutes ~8 Pg of C, the amount comparable to that produced globally by fossil fuel combustion. Thus even a minor increase in decomposition rates of such POM can have a major effect on global C balance.

## Methods

Soil samples were collected from the Long Term Ecological Research (LTER) site at the W. K. Kellogg Biological Station (KBS), Michigan, USA (85°24′ W, 42°24′ N). The soil is Kalamazoo loam (fine-loamy, mixed, mesic, Typic Hapludalf), developed on glacial outwash. The KBS-LTER experiment was established in 1988 (for details on site description, experimental design and research protocols see http://lter.kbs.msu.edu). The three land use and management practices studied are a conventional tillage (chisel-plowed) corn-soybean-wheat rotation with conventional chemical inputs (CT), a certified organic conventional tillage (chisel-plowed) corn-soybean-wheat rotation with red clover cover crop (CT-cover), and native succession vegetation treatment removed from agricultural production in 1989 (NS).

To procure intact soil samples for this study soil blocks (approximately 15 × 15 × 15cm in size) were taken from the top soil layer (i.e., 0–15 cm depth) and dry-sieved. Intact aggregates 4–6.3 mm in size were retained for further analysis^22^. Ten randomly selected aggregates, i.e., intact soil samples, from each treatment were selected. The intact soil samples were scanned, subjected to 120 day incubation, and scanned again.

Scanning was conducted on the bending magnet beam line, station 13-BM-D of the GeoSoilEnvironCARS (GSECARS) at the Advanced Photon Source (APS), Argonne National Laboratory (ANL), IL. Data were collected with the Si (111) double crystal monochromator tuned to 28 keV incident energy, the distance from sample to source was approximately 55 m, and the X-ray doze is estimated to be 1 kGy. Detailed description of the scanning and image analyses procedures are provided by Kravchenko *et al.*^23^. General image analysis tasks were performed using ImageJ and its plug-in tools 3D Viewer^35^ and BoneJ^36^.

**Figure 1:**
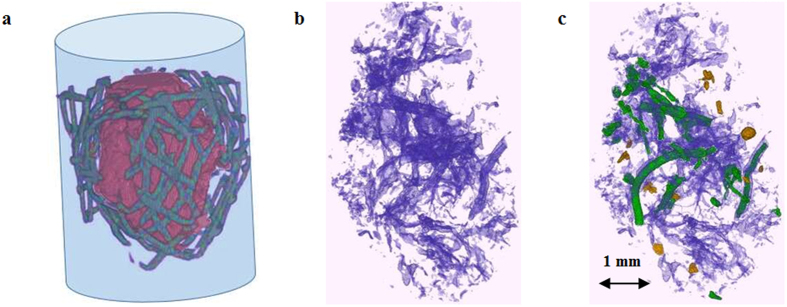
Analysis of μCT images of the studied intact soil samples. (**a**) A setup used for μCT scanning and incubation of the intact soil samples. The sample (brown) is placed in a tube and is held there by means of a plastic mesh (gray) that does not impede air flow. (**b**) Pores with >13 μm equivalent diameter (blue) identified on the μCT 3D image of an intact soil sample. (**c**) Pores with >13 μm equivalent diameter (blue), particulate organic matter (POM) connected by >13 μm pores to the atmosphere (green), and POM not connected to the atmosphere (yellow) identified within an intact soil sample.

**Figure 2:**
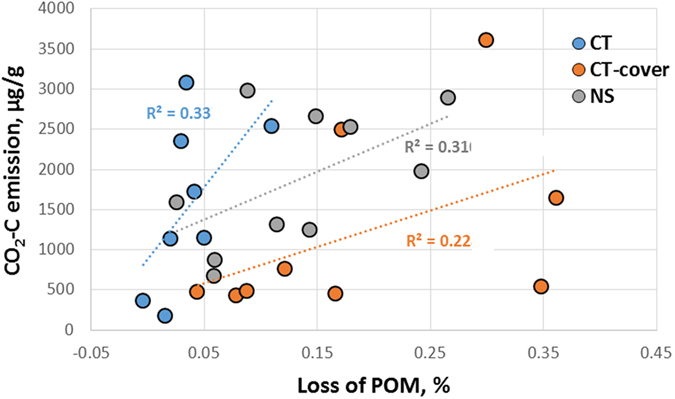
Losses of particulate organic matter (POM) are positively associated with cumulative amount of CO_2_-C emitted during the 120 day incubation. Regressions are statistically significant at 0.1 in CT and NS practices.

**Figure 3:**
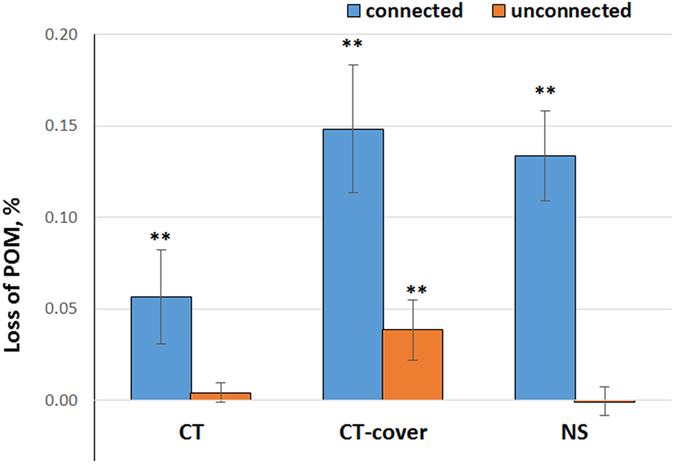
Losses in particulate organic matter (POM) connected and unconnected to the atmosphere by pores >13 μm. Loss of POM is measured as the change in % of POM image voxels of the total number of voxels in the intact soil sample. Bars represent standard errors. Cases when the loss was significantly (p < 0.05) greater than zero are marked by **.

**Figure 4:**
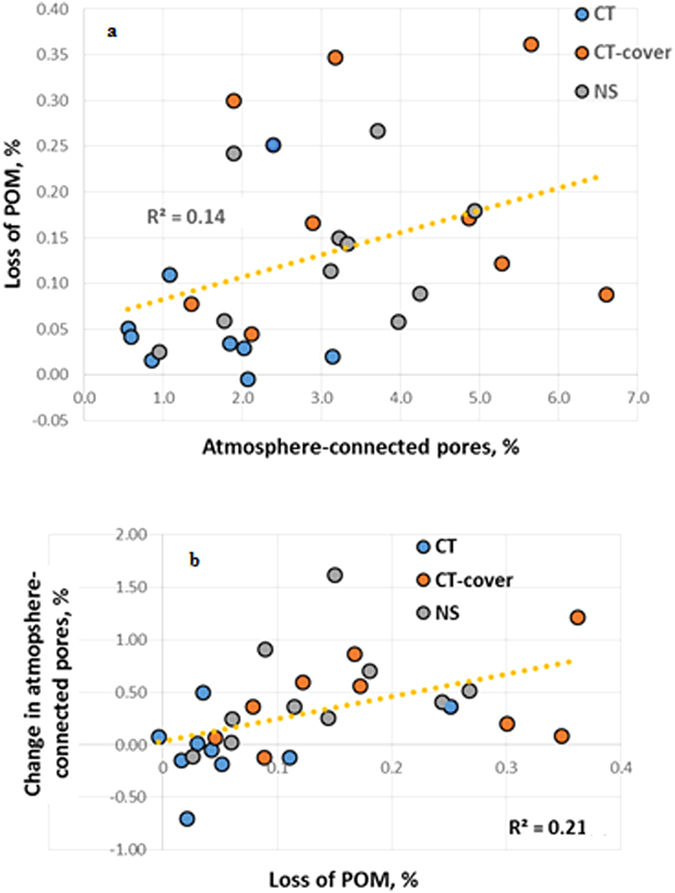
Atmosphere-connected pores with >13 μm equivalent diameter enhance decomposition of particulate organic matter (POM), while greater POM decomposition leads to greater formation of these pores. (**a**) Atmosphere-connected pores (>13 μm) prior to the start of the incubation vs. POM lost during the 120 day incubation. (**b**) POM lost during the incubation vs. the change in atmosphere-connected pores (>13 μm) that took place during the incubation. Pores and changes in pores are measured as the percent of pore voxels from the total number of voxels in the intact soil sample. For both plots the coefficients of determination are calculated based on the data from all three studied practices (p < 0.05).

**Figure 5:**
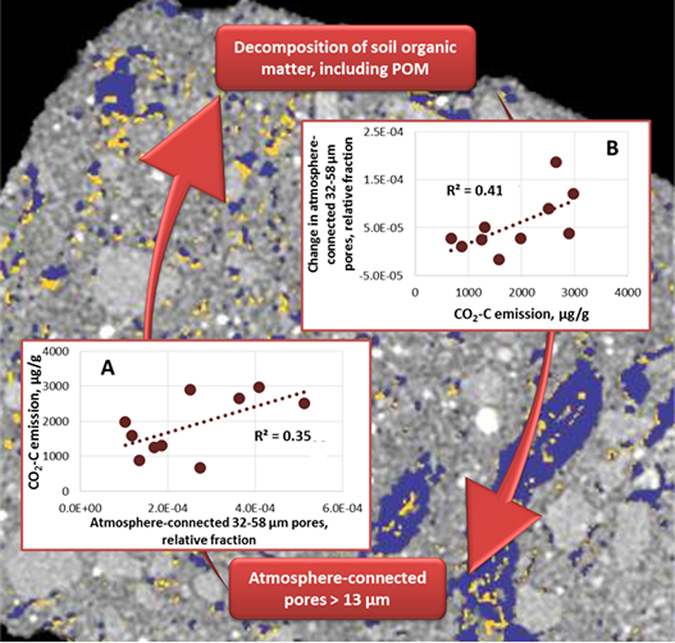
Conceptual model of the positive feedback relationship between soil pores and soil organic matter decomposition. (**a**) The atmosphere-connected 32–58 μm pores were associated with greater cumulative amount of CO_2_-C emitted during the 120 day incubation while (**b**) greater cumulative amount of emitted CO_2_-C increased presence of atmosphere-connected 32–58 μm pores in soil under long-term native vegetation (NS). Coefficients of determination are calculated based on 10 intact soil samples (p < 0.05). On the background is a soil image with initially present >13 μm pores (blue) and additional >13 μm pores that appeared following incubation (yellow).

**Figure 6:**
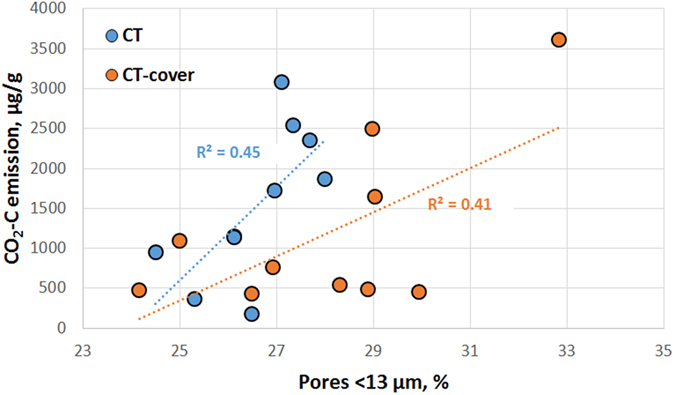
Cumulative amount of CO_2_-C emitted during the 120 day incubation in annually plowed soils of CT and CT-cover practices is positively associated with presence of pores  <13 μm in size. Regressions are statistically significant at 0.05.

Based on the weight of each intact sample and based on its volume determined from the μCT image we calculated total porosity of each sample prior to the incubation. Pores >13 μm were identified on the images with pore/solid material thresholded using indicator kriging approach^37^. Presence of pores was quantitatively described as percent of pore voxels from the total number of soil sample voxels, and is referred to as image-based porosity. Percent of pores <13 μm was determined as the difference between the total volumetric porosity of the sample and its image-based porosity. Note that for accurate determination of the total porosity after the incubation the sample weights would need to be remeasured. That would require taking the aggregates out of the tubes in which they were incubated and unwrapping the protective wire holding them in place (Fig. 1a). Unfortunately, that procedure turned out to be impossible to perform without some losses of soil material, thus accurate determination of the total porosity and <13 μm pores after the incubation could not be achieved. However, due to our careful experimental set-up (Fig. 1a) all studied aggregates remained intact during the entire experiment and none of their post-incubation pore measurements were compromised.

Pore-size distributions were obtained via burn number distribution approach implemented in 3DMA-Rock software^38^. It should be noted that the abundance of pores of different sizes using this approach is represented by relative fractions of median axis voxels of different burn numbers. While an accurate representation of an overall relative frequencies of pores of different sizes, these relative fractions can not be used for estimating the actual volume of pores of different sizes.

POM is commonly defined as the pieces of organic material in soil >0.053 mm in size and is experimentally measured in soil samples based on that definition^39^. POM determination from the images was conducted using the procedure developed by Kravchenko *et al.*^21^. POM was quantified on a volumetric percent basis, i.e., based on the ratio of the number of POM voxels and the total number of voxels in the intact soil sample. Pore characteristics and POM were determined from the images before and after the incubation. The differences in the numbers of voxels of pores of specific sizes and in the numbers of POM voxels in before- and after-incubation images were used to estimate the changes that took place during the incubation.

Each intact soil sample was wrapped in a plastic mesh with 0.5 mm mesh grid and placed in a 3 ml plastic syringe (Ø 10 mm) (BD Franklin Lakes NJ, USA) (Fig. 1a). The purpose of the mesh wrap was to hold the sample suspended inside the syringe without restricting the air flow towards the sample from any of the directions. The average weight of the intact soil samples was 0.26 g with minimum and maximum weights of 0.23 and 0.32 g, respectively. After being placed in the syringes the samples were subjected to μCT scanning. The samples were scanned while air-dry, since it has been demonstrated that scanning has minimal effect on soil microorganisms in dry conditions^40^. Our previous assessments of the effect of scanning on the microbial activities and microbial community structure in intact soil samples of the same size scanned at the same settings demonstrated minimal interference of scanning with microorganisms^23^.

After the first scanning, water content in all intact soil samples was brought to 60% of the total soil porosity. Then the base of each syringe was covered with rubber sleeve stopper, and each syringe was placed in a 10 ml vacutainer (BD Franklin Lakes NJ, USA). Approximately 0.5 ml of water was added to the bottom of each vacutainer to protect soil from drying during the incubation.

The incubations were carried out at 22 °C for 120 days. The CO_2_ emission was measured on the first, second, fourth and eighth days of the incubation and then continued on a weekly basis until the last month of the study, when CO_2_ was measured at two and three week intervals. The CO_2_ measurements were conducted using infrared gas analyzer (LI-820 CO_2_ Analyzer Lincoln, Nebraska, USA). After each sampling, the remaining gas in the headspace was flushed with CO_2_-free air.

After the incubation, prior to the second scanning, the samples were completely air-dried. Thus, both scannings were conducted at the same level of soil moisture, equal to approximately 2–4% for the studied aggregates.

Comparisons between the practices in terms of pore characteristics, POM, and their changes after incubation were conducted using the mixed model approach implemented in the PROC MIXED procedure of SAS (SAS, 2009). Associations between presence of pores of different sizes, amounts of POM and their changes were examined using correlation and linear regression analyses in PROC REG. The regression diagnostic was conducted using Cook’s distance values to examine the influence of individual data points on the regression results and identify outlier; the high influence observations were excluded from the analysis^41^. The significant differences at 0.05 and 0.1 levels are reported.

When the regression results indicated presence of interactions between the management practices and the pore effects, that is, when the regression slopes obtained for different practices differed from each other, the regression analyses of the data for each practice were conducted and reported separately. When no differences in relationships with pores were observed among the three practices, we conducted regression analysis of the entire data set, while still using different symbols for different practices in the figures presenting the regression results. These analyses were conducted using ANCOVA techniques by treating practice as a class variable and pore characteristics as continuous exploratory variables in the PROC MIXED procedure of SAS^42^.

## Additional Information

**How to cite this article**: Kravchenko, A. N. *et al.* Protection of soil carbon within macro-aggregates depends on intra-aggregate pore characteristics. *Sci. Rep.* **5**, 16261; doi: 10.1038/srep16261 (2015).

## Supplementary Material

Supplementary Information

Supplementary Movie 1

## Figures and Tables

**Table 1:**
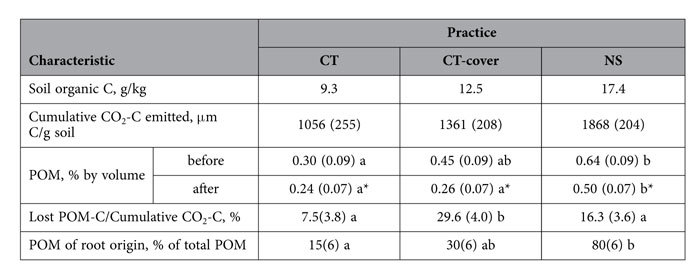
Soil C content of the studied practices along with cumulative amount of CO_2_-C emitted from the intact soil samples during 120 day incubation and particulate organic matter (POM) estimated from X-ray μCT images before and after the incubation.

Standard errors are shown in parentheses. Letters indicate significant differences among the treatments (p < 0.05). Stars in the “after” column for POM indicate significant differences between before and after incubation data within each treatment (p < 0.05). The ratio of POM-C lost to the total amount of emitted CO_2_-C was estimated by assuming POM density of 0.98*10–6 μg/μm3^21^ and C content of POM of 0.279%^18^.

**Table 2:** Pore characteristics of the 4–6 mm intact soil samples from the studied practices (n = 9–10 per practice) before and after 120 day incubations.

Pore characteristic	Practice					
	CT		CT-cover		NS	
Measurements conducted before the incubation						
Total porosity, %	29 (0.9) a		33 (0.9) b		36 (0.9) b	
Pores <13 μm, %	26.6 (0.8) a		28.0 (0.8) a		31.9 (0.8) b	
Measurements conducted before and after the incubation						
	before	after	before	after	before	after
Pores >13 μm, %	3.0 (0.50) a	2.9 (0.55) a	5.3 (0.50) b	6.0 (0.55) b*	4.0 (0.50) ab	4.6 (0.55) ab*
Individual pore size classes, relative fractions						
13–32 μm	0.00035 (0.00003) a	0.00044 (0.00005) a*	0.00050 (0.00006) ab	0.00090 (0.00009) b*	0.00056 (0.00007) b	0.00088 (0.00009) b*
32–58 μm	0.00022 (0.00003)	0.00025 (0.00004)	0.00033 (0.00006)	0.00043 (0.00008)*	0.00035 (0.00005)	0.00040 (0.00006)*
58–84 μm	0.00007 (0.000012)	0.00008 (0.000015)	0.00012 (0.000024)	0.00014 (0.000028)*	0.00012 (0.000014)	0.00012 (0.000016)
>84 μm	0.000048 (0.000009) a	0.000048 (0.000011) a	0.000126 (0.000026) b	0.000125 (0.000022) b	0.000075 (0.000011) a	0.000073 (0.000010) ab

Standard errors are shown in parentheses. Letters indicate significant differences between the practices within before and after groups (p < 0.05). Stars in the “after” columns indicate significant differences between before and after incubation data within each practice (p < 0.05). Accurate assessments of intact soil sample mass were possible only prior to the incubation, thus total porosity and presence of pores <13 μm were determined only for the initial state of the samples.
