# Supplementary material for: Protection of soil carbon within macro-aggregates depends on intra-aggregate pore characteristics
Source: Sci Rep. 2015 Nov 6;5:16261. doi: 10.1038/srep16261 (PMC4635377; doi:10.1038/srep16261)
Supplement: Supplementary Information [file srep16261-s1.pdf]

Supplementary Video 1 Legend for the manuscript:

Title: Protection of soil carbon within macro-aggregates depends on intra-aggregate pore characteristics

Authors: Alexandra N. Kravchenko, Wakene C. Negassa Andrey K. Guber, Mark L. Rivers

Supplementary Video 1. An illustration of an intact soil sample subjected to 3D X-ray computed tomography scanning. Visible are pores with >13 μm equivalent diameter (blue), particulate organic matter (POM) connected by >13 μm pores to the atmosphere (green), and POM not connected to the atmosphere (yellow) identified within an intact soil sample.
